# Defining and understanding the “extra‐corporeal membrane oxygenation gap” in the veno‐venous configuration: Timing and causes of death

**DOI:** 10.1111/aor.14058

**Published:** 2021-09-07

**Authors:** Samuel Heuts, Maged Makhoul, Abdulrahman N. Mansouri, Fabio Silvio Taccone, Amir Obeid, Mirko Belliato, Lars Mikael Broman, Maximilian Malfertheiner, Paolo Meani, Giuseppe Maria Raffa, Thijs Delnoij, Jos Maessen, Gil Bolotin, Roberto Lorusso

**Affiliations:** ^1^ Department of Cardiothoracic Surgery Maastricht University Medical Center+ Maastricht The Netherlands; ^2^ Cardiac Surgery Unit Rambam Medical Centre Haifa Israel; ^3^ Department of Intensive Care Medicine Université Libre de Bruxelles, Clinique Universitaire de Bruxelles (CUB) Erasme Brussels Belgium; ^4^ ECMO Unit IRCCS Monzino Heart Centre Milan Italy; ^5^ ECMO Centre Karolinska Karolinska University Hospital Stockholm Sweden; ^6^ Department of Internal Medicine University of Regensburg Regensburg Germany; ^7^ Department of Cardiothoracic, Vascular Anesthesia and Intensive Care IRCCS Policlinico San Donato Milan Italy; ^8^ Department for the Treatment and Study of Cardiothoracic Diseases and Cardiothoracic Transplantation ISMETT‐IRCCS Palermo Italy; ^9^ Department of Cardiology Maastricht University Medical Center+ Maastricht The Netherlands; ^10^ Intensive Care Department Maastricht University Medical Center+ Maastricht The Netherlands; ^11^ Cardiovascular Research Institute Maastricht (CARIM) Maastricht University Maastricht The Netherlands

**Keywords:** ECMO, complications, extracorporeal membrane oxygenation, mortality, veno‐venous

## Abstract

In‐hospital mortality of adult veno‐venous extracorporeal membrane oxygenation (V‐V ECMO) patients remains invariably high. However, little is known regarding timing and causes of in‐hospital death, either on‐ECMO or after weaning. The current review aims to investigate the timing and causes of death of adult patients during hospital admittance for V‐V ECMO, and to define the *V‐V ECMO gap*, which is represented by the patients that are successfully weaned of ECMO but still die during hospital stay. A systematic search was performed using electronic MEDLINE and EMBASE databases through PubMed. Studies reporting on adult V‐V ECMO patients from January 2006 to December 2020 were screened. Studies that did not report on at least on‐ECMO mortality and discharge rate were excluded from analysis as they could not provide the required information regarding the proposed V‐V ECMO‐gap. Mortality rates on‐ECMO and after weaning, as well as weaning and discharge rates, were analyzed as primary outcomes. Secondary outcomes were the causes of death and complications. Initially, 35 studies were finally included in this review. Merely 24 of these studies (comprising 975 patients) reported on prespecified V‐V ECMO outcomes (on‐ECMO mortality and discharge rate). Mortality on V‐V ECMO support was 27.8% (95% confidence interval (CI) 22.5%‐33.2%), whereas mortality after successful weaning was 12.7% (95% CI 8.8%‐16.6%, defining the *V‐V ECMO gap*). 72.2% of patients (95% CI 66.8%‐77.5%) were weaned successfully from support and 56.8% (95% CI 49.9%‐63.8%) of patients were discharged from hospital. The most common causes of death *on ECMO* were multiple organ failure, bleeding, and sepsis. Most common causes of death *after weaning* were multiorgan failure and sepsis. Although the majority of patients are weaned successfully from V‐V ECMO support, a significant proportion of subjects still die during hospital stay, defining the *V‐V ECMO gap*. Overall, timing and causes of death are poorly reported in current literature. Future studies on V‐V ECMO should describe morbidity and mortality outcomes in more detail in relation to the timing of the events, to improve patient management, due to enhanced understanding of the clinical course.

## Graphical abstract

Graphical abstract present the V‐V ECMO gap. In 24 studies comprising 975 patients, on‐ECMO mortality was 27.8%, while 72.2% of patients were weaned successfully. Still 12.7% of patients deceased after weaning, defining the proposed V‐V ECMO gap.

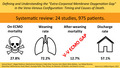

## 1 INTRODUCTION

Extracorporeal membrane oxygenation (ECMO) was first used successfully in the beginning of the 1970s.^1^ Since then, almost 15 years passed until the first acceptable survival rates (49%) were published in 1986.^2^ In the following years, a remarkable improvement was observed in ECMO applications, mainly in neonates and children,^3^, ^4^ which encouraged its use in adults as well.^5^ In critically ill patients, ECMO can provide temporary cardiopulmonary support, separately or in combination, providing the heart and the lungs the time needed to recover from an acute severe insult. Different ECMO configurations are used for different indications. In case of respiratory failure, where the ability of lungs to exchange gas is severely diminished, a veno‐venous ECMO (V‐V ECMO) mode is generally applied.

Previous and recent multicenter, randomized controlled trials showed a trend towards reduced mortality in favor of V‐V ECMO over conventional treatment.^6^, ^7^ Furthermore, in a recently published meta‐analysis of these trials, 60‐day mortality was significantly lower in the V‐V ECMO group.^8^

Although mortality rates in V‐V ECMO are lower compared with other ECMO modalities, a substantial quote of patients still die during hospital admittance.^9^ According to the Extracorporeal Life Support Organization (ELSO), more than 75 000 ECMO runs for respiratory compromise have been performed in more than 450 centers (as of July 2021). Around 40% of these runs are for *neonatal* respiratory disease, with a reported survival‐to‐discharge rate of 73%, while *adult* respiratory V‐V ECMO runs also comprise around 40% of the V‐V ECMO runs, with a reported survival‐to‐discharge rate of 59%.^10^

Many published V‐V ECMO series describe mortality rates. However, timing and causes of death are rarely reported and often not specifically related to mortality and survival. As such, mortality and timing of mortality (either on‐ECMO or after weaning) of V‐V ECMO patients is still not fully elucidated. Although less well pronounced as in V‐A ECMO,^11^ we still observe a relatively large group of patients that decease despite successful weaning from ECLS in V‐V ECMO patients. In a previously published review of V‐A ECMO patients by our research group,^11^ this discrepancy was identified as the *ECMO gap*, which can also be applied to patients undergoing V‐V ECMO: *the V‐V ECMO gap*.

As we are in the midst of an unprecedented COVID‐19 pandemic, and V‐V ECMO is increasingly used to support this specific subgroup of patients, it is imperative to comprehend this aspect of ECMO outcomes, to potentially enhance patient’s survival, and promote counteractions meant to address the adverse events. Therefore, the primary aim of the current systematic review was to establish actual mortality rates, particularly regarding the timing, whether on V‐V ECMO or after successful weaning of V‐V ECMO (which defines the *V‐V ECMO gap*), and the cause of death as well as the complications.

## 2 METHODS AND MATERIALS

### 2.1 Literature search strategy

A systematic search was performed using MEDLINE and EMBASE electronic databases. We adhered to the PRISMA guidelines for reporting in systematic reviews and meta‐analyses.^12^ The following search terms were used: extracorporeal life support, ECLS, ECMO, and V‐V ECMO. Additionally, reference lists of the prescreened studies were manually checked for additional eligible studies.

With the advent of the COVID‐19 pandemic in 2020, V‐V ECMO therapy has gained increasing attention as it has been successfully applied in these severely ill patients with acute respiratory distress syndrome (ARDS).^13^ We are still in the midst of this pandemic, and many studies and registries are ongoing. Together with the fact that COVID‐19 is such a distinct disease with unique features and pulmonary consequences,^14^ we decided to exclude this patient population to perform a separate analysis in a future study. Studies published between January 2006 and December 2020 were eligible for inclusion. Articles with a study population under 18 years of age were regarded as pediatric and not considered for inclusion. Of note, the study was registered in PROSPERO (CRD 42020140971, registration date October 8th, 2019).^15^

### 2.2 Study criteria

Due to the emergent nature of the condition and the expected small amount of randomized and prospective data, we considered all randomized, prospective, observational, and retrospective studies and case series for inclusion. Editorials, commentaries, letters to editor, opinion articles, reviews, or meeting abstracts were excluded. To reduce and limit the risk of imprecision and publication bias, case reports were excluded as well. Studies encompassing less than five patients were also excluded. Animal studies and non‐English studies were not considered. Patient cohorts under the age of 18 were deemed pediatric and excluded. When there were mixed populations (pediatric and adult), the study was only considered for inclusion when data were analyzed separately for adults and children. All studies describing non‐V‐V ECMO or other ECLS support modalities were excluded. When a study reported on a combination of V‐V and V‐A data, the study was only considered for inclusion if V‐V ECMO outcomes were analyzed and provided separately. When we found multiple publications by the same group describing a growing population, only the most recent study was considered for inclusion. Finally, studies that did not report on at least on‐ ECMO mortality and discharge rate were excluded from analysis as they could not provide the required information for the proposed ECMO‐ gap.

### 2.3 Data extraction

Two independent researchers with extensive expertise in statistics and epidemiology extracted the following outcomes from each publication: year of publication, number of patients, on‐ECMO mortality, weaning rate, in‐hospital mortality after weaning, and discharge rate. If available, the following additional data were extracted: timing and cause of death on‐ECMO, in‐hospital cause of death after weaning and in‐hospital complications.

### 2.4 End‐point definition

The primary outcome of the review was the reported rates of mortality on‐ECMO and after weaning during the ECMO‐related hospitalization. Secondary outcomes were the causes of death either on‐V‐V ECMO or after weaning, rate of hospital discharge, and in‐hospital complications of V‐V ECMO patients. As only a few studies described causes of death on‐ECMO and after weaning specifically, they formed the base of the current review.

We defined the *V‐V ECMO gap* as follows: the difference between the rate of patients who were successfully weaned from V‐V ECMO and the rate of patients who were finally discharged at the end of the V‐V ECMO‐related hospital admission, that is patients died after successful ECMO weaning.

### 2.5 Data synthesis

Pooling of survival and weaning rates was performed to elucidate the V‐V ECMO gap. Based on the study sample size and the distribution of data, every separate study was assigned a certain weight. Random‐effect models were used given the expected differences between studies. Survival and weaning data were reported as means with their corresponding 95% confidence intervals (CI). Heterogeneity was tested using the *I* ^2^‐test for heterogeneity, in which a result of >50%, in conjunction with a *P* value < .10 was considered significant. Due to the expected relatively low methodological quality of the studies and a variety of indications for V‐V ECMO in a differing patient population, substantial heterogeneity was expected and results should therefore be interpreted with caution. Although pooling of data is generally not advised in presence of such levels of heterogeneity, we do consider the outcomes to be relevant, as the mere heterogeneity itself also is part of the V‐V ECMO gap and the mis‐ and underreporting in V‐V ECMO studies. Complications and causes of death were reported as ranges. A freely available software package (OpenMetaAnalyst, http://www.cebm.brown.edu/openmeta) was used for data synthesis.

## 3 RESULTS

### 3.1 Included studies

**FIGURE 1:**
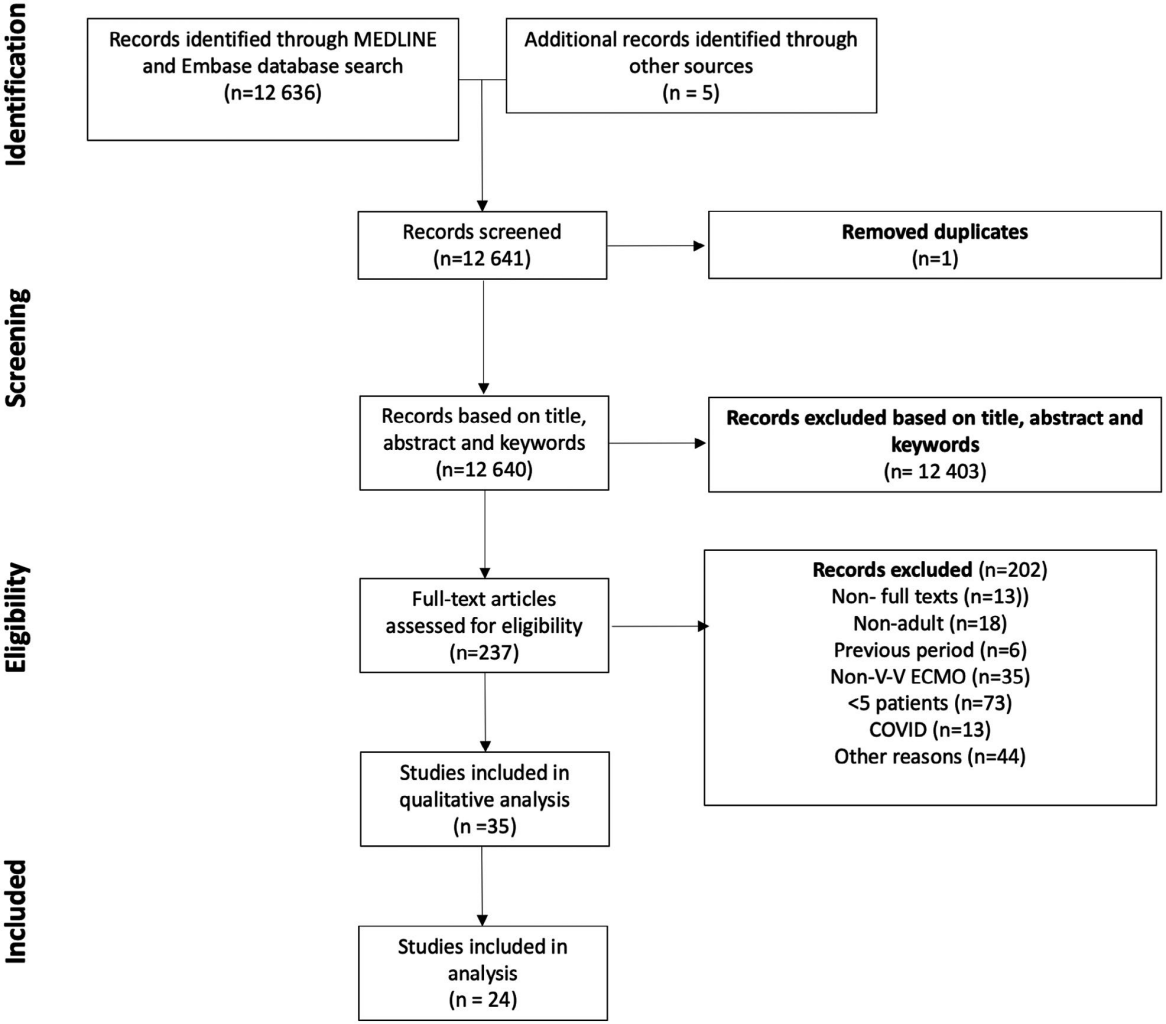
Study selection procedure shown in a PRISMA flow diagram. COVID, coronavirus disease; V‐V ECMO, veno‐venous extracorporeal membrane oxygenation

The literature search generated 12 636 hits, 5 studies were added using other sources, one duplicate was excluded. In total, 12 403 articles were excluded during screening based on title, abstract, and keywords. Then, 202 publications were excluded based on full‐text reading for a variety of reasons (see Figure 1, PRISMA flowchart). Eventually, 35 articles were included (Supplementary Data 1). Of these 35 selected articles (Table S1), only 24 (69%, Table 1) described in‐hospital outcomes (on‐ECMO mortality, weaning rate, in‐hospital mortality after weaning, and discharge rate) in detail, enabling the evaluation and appraisal of the *V‐V ECMO gap*. Therefore, these 24 articles were used for further analysis of the *V‐V ECMO gap*.^16^, ^17^, ^18^, ^19^, ^20^, ^21^, ^22^, ^23^, ^24^, ^25^, ^26^, ^27^, ^28^, ^29^, ^30^, ^31^, ^32^, ^33^, ^34^, ^35^, ^36^, ^37^, ^38^, ^39^ Of note, two separate studies were included by the same author^37^, ^38^ describing distinct study groups (patients with trauma and nontrauma). The selected 24 articles comprised a total of 975 patients. The number of patients in each article varied between 8 and 116.

**TABLE 1:** Study characteristics and V‐V ECMO outcomes, including on‐ECMO mortality, weaning rate, and after weaning mortality rate, and discharge rate

Author	Year	Total number of patients	On ‐ECMO mortality (n)	%	95% CI	Weaning rate (n)	%	95% CI	Mortality after weaning (n)	%	95% CI	Discharge rate (n)	%	95% CI
Beiderlinden*	2006	32	5	15.6	3.0‐28.2	27	84.4	71.8‐97.0	10	31.2	15.2‐47.3	17	53.1	35.8‐70.4
Bermudez*	2010	11	3	27.3	1.0‐53.6	8	72.7	46.4‐99.0	2	18.2	0‐41.0	6	54.5	25.1‐84.0
Bonacchi	2011	30	5	16.7	3.3‐30.0	25	83.3	70.0‐96.7	3	10.0	0‐20.7	22	73.3	57.5‐89.2
Buchner	2018	13	1	7.7	0‐22.2	12	92.3	77.8‐100	1	7.7	0‐22.2	11	84.6	65.0‐100
Cheng*	2016	116	32	27.6	19.5‐35.7	84	72.4	64.3‐80.5	22	19.0	11.8‐26.1	62	53.4	44.4‐62.5
Chimot*	2013	52	13	25.0	13.2‐36.8	39	75.0	63.2‐86.8	12	23.1	11.6‐34.5	27	51.9	38.3‐65.6
Chiu	2015	65	28	43.1	31.0‐55.1	37	56.9	44.9‐69.0	6	9.2	2.2‐16.3	31	47.7	35.6‐59.8
Hong*	2013	18	1	5.6	0‐16.1	17	94.4	83.9‐100	4	22.2	3.0‐41.4	13	72.2	51.5‐92.9
Kon	2015	55	13	23.6	12.4‐34.9	42	76.4	65.1‐87.6	22	40.0	27.1‐52.9	20	36.4	23.7‐49.1
Kutlesa*	2017	40	11	27.5	13.7‐41.3	29	72.5	58.7‐86.3	4	10.0	0.7‐19.3	25	62.5	47.5‐77.5
Lee*	2015	45	24	53.3	38.8‐67.9	21	46.7	32.1‐61.2	13	28.9	15.6‐42.1	8	17.8	6.6‐28.9
Messai	2013	17	7	41.2	17.8‐64.6	10	58.8	35.4‐82.2	1	5.9	0‐17.1	9	52.9	29.2‐76.7
Munshi	2017	57	18	31.6	19.5‐43.6	39	68.4	56.4‐80.5	0	0.9	0‐3.2	39	68.4	56.4‐80.5
Nakamura*	2013	11	4	36.4	7.9‐64.8	7	63.6	35.2‐92.1	1	9.1	0‐26.1	6	63.6	35.2‐92.1
Ng*	2014	31	6	19.4	5.4‐33.3	25	80.6	66.7‐94.6	1	3.2	0‐9.4	24	77.4	62.7‐92.1
Noah*	2011	69	10	14.5	6.2‐22.8	59	85.5	77.2‐93.8	8	11.6	4.0‐19.1	51	73.9	63.6‐84.3
Pappalardo*	2013	60	19	31.7	19.9‐43.4	41	68.3	56.6‐80.1	0	0.8	0‐3.1	41	68.3	56.6‐80.1
Reeb*	2017	8	3	37.5	4.0‐71.0	5	62.5	29.0‐69.0	1	12.5	0‐35.4	4	50.0	15.4‐84.6
Roch*	2014	77	38	49.4	38.2‐60.5	39	50.6	39.5‐61.8	6	7.8	1.8‐13.8	33	42.9	31.8‐53.9
Song*	2016	13	4	30.8	5.7‐55.9	9	69.2	44.1‐94.3	2	15.4	0‐35.0	7	53.8	26.7‐80.0
Voelker	2015	18	7	38.9	16.4‐61.4	11	61.1	38.6‐83.6	0	2.6	0‐9.8	11	61.1	38.6‐83.6
Wohlfarth*	2014	11	4	36.4	7.9‐64.8	7	63.6	35.2‐92.1	3	27.3	1.0‐53.6	4	36.4	7.9‐64.8
Wu*	2014	20	4	20.0	2.5‐37.5	16	80.0	62.5‐97.5	2	10.0	0‐23.1	14	70.0	49.9‐90.1
Wu*	2017	106	35	33.0	24.1‐42.0	71	67.0	58.0‐75.9	21	19.8	12.2‐27.4	50	47.2	37.7‐56.7
n = 24	Total	975	295	27.8	22.5‐33.2	680	72.2	66.8‐77.5	145	12.7	8.8‐16.6	535	57.1	50.2‐64.0

Abbreviations: ECMO, extracorporeal membrane oxygenation; NR, not reported.

* Studies that report on causes of death.

### 3.2 Mortality rates on‐ECMO and after weaning, weaning, and discharge

All parameters considered for calculation of the V‐V ECMO gap (on‐ECMO mortality, weaning rate, after weaning mortality, discharge rate) were reported by all 24 studies and presented in Table 1.

On‐ECMO mortality occurred in 295/975 patients with a pooled mortality rate of 27.8% (95% CI 22.5%‐33.2%), varying between 5.6% and 53.3%. Heterogeneity was significant (*I* ^2^ = 71.2%, *P* < .001).

**FIGURE 2:**
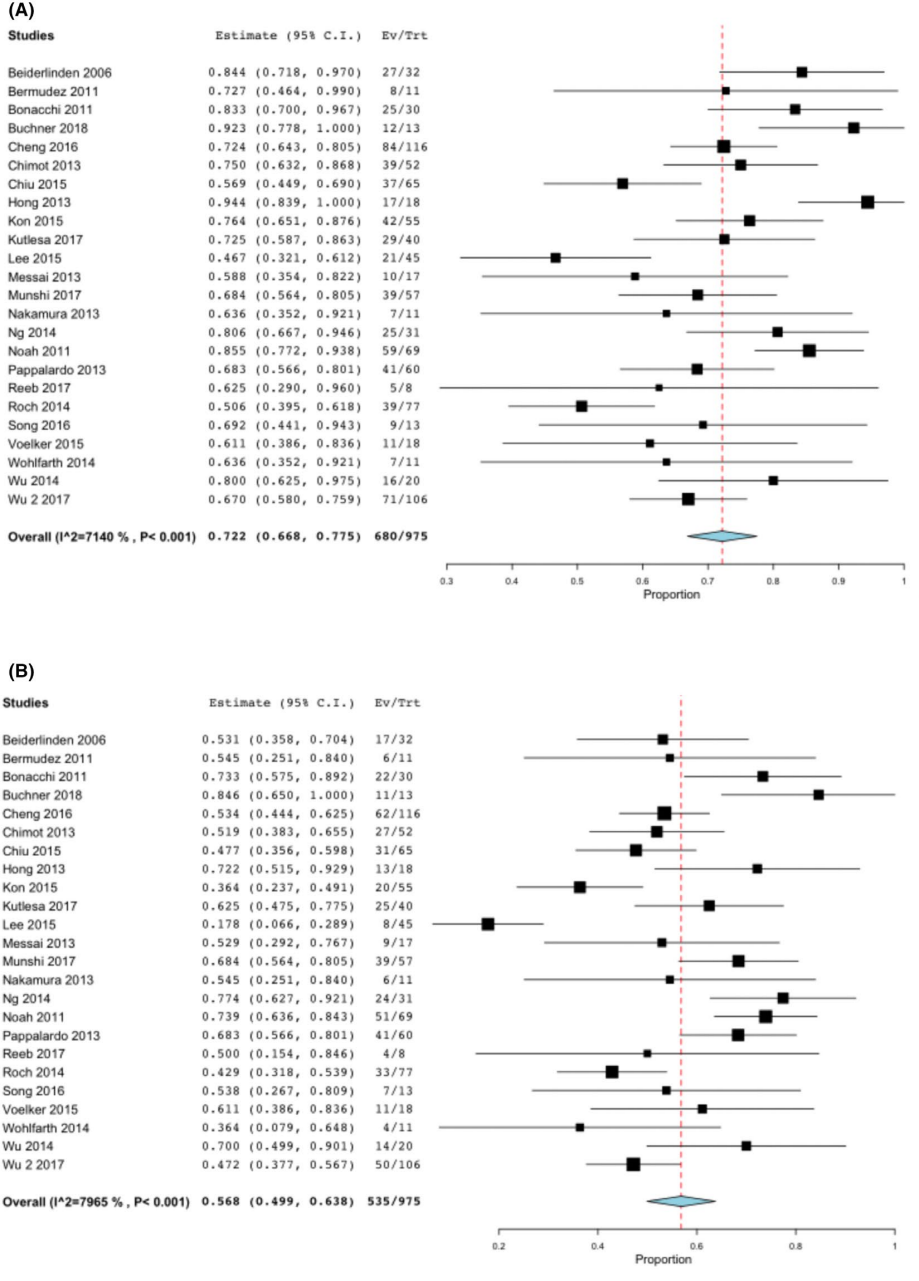
Forest plots depicting (A) weaning rate from veno‐venous extracorporeal membrane oxygenation and (B) hospital discharge rate. CI, confidence interval [Color figure can be viewed at wileyonlinelibrary.com]

680/975 patients were successfully weaned from V‐V ECMO, resulting in a pooled weaning rate of 72.2% (95% CI 66.8%‐77.5%), ranging from 46.7% to 94.4%. Again, significant heterogeneity was noted (*I* ^2^ = 71.4%, *P* < .001). Figure 2A demonstrates weaning rate graphically in a forest plot.

Although 72.2% of patients were successfully weaned, 145/975 patients still died after weaning, before discharge (pooled after weaning mortality rate of 12.7%, 95% CI 8.8%‐16.6%), ranging from 0% to 40.0%. As such, this rate of 12.7% represents our predefined V‐V ECMO gap. Of note, heterogeneity of these results was significant (*I* ^2^ = 82.6%, *P* < .001). Only six studies reported on the duration of time between weaning and mortality (ranging from 1 to 62 days),^22^, ^28^, ^32^, ^33^, ^34^, ^39^ impairing analysis of potential associations.

Eventually, of the whole patient group, 535/975 patients were discharged from hospital, leading to an overall pooled discharge rate of 56.8% (95% CI 49.9%‐63.8%). Significant heterogeneity was demonstrated by *I* ^2^ = 79.7%, *P* < .001. Figure 2B graphically depicts the hospital discharge rate in a forest plot.

### 3.3 Causes of death

Causes of death were specified and related to mortality in 17/24 studies (71%, marked with an * in Table 1). These 17 studies encompass 720 patients. In this subgroup of studies, 216 patients (pooled on‐ECMO mortality rate 27.8% (95% CI 21.3%‐34.4%)) died while on‐ECMO. After weaning mortality rate was 14.5% (95% CI 9.2%‐19.7%) in this subgroup.

Table 2 presents the causes of death in these studies. Most common cause of death on‐ECMO was multiple organ failure (MOF) (ranging from 0% to 89%), followed by bleeding (ranging from 0% to 100%) and sepsis/infection (ranging from 0% to 100%). The most common cause of death in‐hospital after weaning of V‐V ECMO was MOF (ranging from 0% to 100%), and sepsis/infection (ranging from 0% to 100%), although it should be noted that cause of death was unknown in a significant number of cases in this setting. Cerebral hemorrhage as a cause of death was found on‐ECMO (ranging from 0%‐70%) as well as after weaning (ranging from 0% to 50%).

**TABLE 2:** Causes of death on‐ECMO and after weaning

Author	Year	Cause of death on‐ECMO (n, %)	Cause of death after weaning (n, %)
Beiderlinden	2006	MOF (n = 4, 80%)	Neurological (n = 1, 10%)
			Sepsis/infection (n = 2, 20%)
		Cerebral hemorrhage (n = 1, 20%)	Unknown (n = 7, 70%)
Bermudez	2010	MOF (n = 2, 67%)	Cerebral hemorrhage (n = 1, 50%)
		Bleeding (n = 1, 33%)	Sepsis (n = 1, 50%)
Cheng	2016	MOF (n = 22, 69%)	MOF (n = 22, 100%)
		Cerebral hemorrhage (n = 4, 13%)	
		Bleeding (n = 6, 19%)	
Chimot	2013	MOF (n = 8, 62%)	Unknown (n = 12, 100%)
		Neurological (n = 3, 23%)	
		Air‐embolism (n = 1, 8%)	
		Device‐related (n = 1, 8%)	
Hong	2013	Bleeding (n = 1, 100%)	Persistent respiratory failure (n = 4, 100%)
Kutlesa	2017	Sepsis (n = 9, 82%)	Sepsis (n = 1, 25%)
		Pulmonary embolism (n = 1, 9%)	Cerebral hemorrhage (n = 2, 50%)
		Myocardial infarction (n = 1, 9%)	Pulmonary embolism (n = 1, 25%)
Lee	2015	Cerebral hemorrhage (n = 1, 4%)	Sepsis/infection (n = 11, 85%)
		Bleeding (n = 14, 58%)	Unknown (2, 15%)
		Device‐related (n = 2, 8%)	
		Thrombo‐embolism (n = 2, 8%)	
		Cardiac arrest (n = 5, 21%)	
Noah	2011	MOF (n = 1, 10%)	MOF (n = 2, 25%)
		Cerebral hemorrhage (n = 7, 70%)	Sepsis (n = 1, 13%)
			Cerebral hemorrhage (n = 1, 13%)
		Bleeding (n = 1, 10%)	Bleeding (n = 1, 13%)
		Cardiac arrest (n = 1, 10%)	Persistent respiratory failure (n = 2, 25%)
			Rhabdomyolysis (n = 1, 13%)
Nakamura	2013	Cardiac failure (n = 1, 33%)	Persistent respiratory failure (n = 1, 100%)
		Bowel ischemia (n = 2, 67%)	
Ng	2014	Device‐related (n = 2, 33%)	Vascular injury (n = 1, 100%)
		DIC (n = 1, 17%)	
		Thrombo‐embolism (n = 1, 17%)	
		Cerebral hemorrhage (n = 1, 17%)	
		Unknown (n = 1, 17%)	
Pappalardo	2013	MOF (n = 10, 53%)	–
		Sepsis (n = 5, 26%)	
		Unknown (n = 4, 21%)	
Reeb	2017	MOF (n = 2, 67%)	Sepsis (1, 100%)
		Arrhythmia (n = 1, 33%)	
Roch	2014	MOF (n = 34, 89%)	MOF (n = 6, 100%)
		Cerebral hemorrhage (n = 2, 5%)	
		Bleeding (n = 2, 5%)	
Song	2018	Sepsis (n = 4, 100%)	Sepsis (n = 2, 100%)
Wohlfarth	2014	MOF (n = 4, 100%)	Unknown (n = 3, 100%)
Wu	2014	Bleeding (n = 3, 75%)	Sepsis (n = 2, 100%)
		Sepsis (n = 1, 25%)	
Wu	2017	MOF (n = 28, 80%)	MOF (n = 21, 100%)
		Cerebral hemorrhage (n = 2, 6%)	
		Bleeding (n = 5, 14%)	

Abbreviations: DIC, diffuse intravascular coagulation; ECMO, extracorporeal membrane oxygenation; MOF, multiple organ failure.

### 3.4 Complications

Table 3 presents the different complications and the associated complication rate specified per study. Unfortunately, timing of complications could not be retrieved.

**TABLE 3:** In‐hospital complications

Author	Year	Complication	n	%
Bermudez	2010	Neurological	1	9
		Sepsis	1	9
		Cannula related	3	27
Bonacchi	2011	Bleeding	5	17
Buchner	2018	Renal	6	46
		Neurological	1	8
		Bleeding	9	69
		Respiratory	8	62
Cheng	2016	Bleeding	10	9
		Sepsis	46	40
Chimot	2013	Hematological	11	21
		Bleeding	9	17
		Cannula related	4	8
		Other	5	10
		Renal	2	4
		Sepsis	2	4
Hong	2013	Cannula related	1	6
		Bleeding	3	17
		Other	1	6
Kon	2015	Bleeding	21	38
		Hematological	6	11
		Neurological	4	7
Kutlesa	2017	Cannula related	2	5
		Bleeding	7	18
		Renal	16	40
		Respiratory	17	43
		Hematological	16	40
Lee	2015	Bleeding	22	49
		Cannula related	20	44
		Mechanical	2	4
Munshi		Respiratory	5	9
		Leg ischemia	1	2
		Bleeding	1	2
		Hematological	4	7
Ng	2014	Bleeding	1	3
		Respiratory	1	3
		Mechanical	3	10
Noah	2011	Bleeding	23	24
		Other	6	6
		Cannula related	3	3
		Respiratory	4	4
		Hematological	1	1
Pappalardo	2013	Cannula related	5	8
		Other	4	7
Roch	2014	Bleeding	25	33
Wohlfarth	2014	Bleeding	6	54.6
		Leg ischemia	2	18
Wu	2017	Bleeding	6	6

## 4 DISCUSSION

Mortality in V‐V ECMO patients remains high, in spite of an increase in technology, knowledge and experience regarding patient selection and ECMO management.^6^, ^7^ Although there is a substantial increase in ECMO publications, detailed reports on in‐hospital mortality, timing, and cause of death have been poorly provided. Indeed, little is known on timing of death, particularly in regard to on‐ECMO or after weaning mortality. In the current systematic review, it is shown that, although the majority of the patients are generally weaned from V‐V ECMO, still a relatively high percentage of these patients die during hospital stay. We defined this discrepancy between weaning and in‐hospital mortality as the *V‐V ECMO gap*, encompassing almost 13% of adult V‐V ECMO patients.

In the current systematic review, initially 35 studies were included. The small number of selected studies, despite an extensive literature search, underline how rarely the kind of information we were interested in, is actually poorly reported. To define the V‐V ECMO gap, we focused on the percentage of patients who, despite a successful ECMO run and weaning, died during hospital stay (death despite successful ECMO weaning). This condition is certainly of great interest based on the favorable support and likely improved lung function. However, to define the *V‐V ECMO gap* correctly, at least the following outcomes needed to be reported: mortality on‐ECMO, weaning rate, in‐hospital mortality after weaning and discharge rate. Disappointingly, only 24 of 35 studies reported on these relevant outcomes. This finding alone already demonstrates an additional *ECMO gap* in adequately and uniformly reporting on outcomes in ECMO research.

To elucidate the reasons and differences for and between on‐ECMO and after weaning mortality, causes of death were evaluated as well. MOF seems to play the most important role in mortality causes on‐ECMO and after weaning. However, MOF is a widely used diagnosis in ECMO research and can be difficult to interpret due to the heterogeneity of its definition and multifactorial genesis. In many ventilated patients, MOF is evident in the pre‐ECMO phase already, as multiple organs are involved in various types and degrees of failure.^40^ Extensive MOF, which can be graded using several scoring systems, can even be a contraindication for V‐V ECMO due to its futile prognosis.^21^ Therefore, adequate patient selection and improved timing of V‐V ECMO application could help to lower the mortality rate of this patient group.^40^

The question remains, why a relatively high percentage of patients still die after successful weaning from V‐V ECMO. We can only hypothesize that this patient group has been weaned from V‐V ECMO support too early, most likely, or with a suboptimal weaning strategy or postweaning management. Differences and timing in weaning strategies of V‐V ECMO are unfortunately rather based on expert opinion than on strong clinical evidence. These strategies vary in blood flow lowering, carbon dioxide level monitoring, radiological (X‐ray) improvement, post‐ECMO respiratory/ventilatory management, and other clinical assessments or treatments.^41^

Bleeding was another important cause of death, on‐ECMO and after weaning. Although cannulas in the V‐V configuration are usually implanted percutaneously in the venous system, bleeding complications remain high. The main source of bleeding usually tends to be the cannulation site.^42^ In addition, spontaneous bleeding can be induced by anticoagulation therapy or intervened coagulation disorders. By itself, anticoagulation therapy can primarily cause bleeding, but secondary heparin‐induced thrombocytopenia can initiate bleeding as well.^43^ These circumstances potentially result in a vicious circle of bleeding, consumption of coagulation factors, hypovolemia, vasoplegia, and MOF. Although intuitive, regular, and close monitoring of activated partial thromboplastin (aPTT) levels is imperative in these patients, as unstable aPTT levels are an independent predictor of excessive blood loss and mortality.^42^

Sepsis remains an important contributor to survival as well. Patients on V‐V ECMO are more prone to contract bloodstream infections and subsequent sepsis due to the direct contact of the cannulas and the blood vessels. Although direct bloodstream infections occur less frequently after weaning, patient generally require prolonged ventilation, either through a tracheal tube or tracheostomy, making them more prone to ventilator‐associated pneumonia and subsequent pneumosepsis. Close monitoring of blood cultures on‐ECMO and early initiation of (prophylactic) antibiotic treatment in suspected pneumonia in this fragile patient group is, therefore, mandatory.

Once neurological complications occur, generally less than 25% of patients survive to hospital discharge.^44^ Remarkably, in this review, the incidence of on‐ECMO and after weaning neurological causes of death appeared relatively low, particularly if compared with large registry‐based studies.^45^ In this study, cerebral complications were predominantly caused by cerebral hemorrhage (42%) followed by brain death (24%) and stroke (20%). Possibly, underreporting of neurological complications in the studies included in this review is due to a (mis)classification of these complications as bleeding events, given the high incidence of earlier reported cerebral hemorrhages.^45^ Still, the EOLIA‐trial demonstrated a cerebral injury rate of only 2% (ischemic and hemorrhagic stroke) in V‐V ECMO patients, although the lack of post‐mortem examination might have underestimated the actual occurrence.^7^ These findings highlight the importance of a more careful anticoagulation management, or improved coagulation disorder treatment/prevention, not underestimating an improved patient selection and ECMO management. Finally, as a substantial proportion of causes of death after weaning remained unknown, a significant number of these cases could be contributed to neurological causes, but the rather high percentage of non‐described events represents another example of limited reporting in patients on ECMO.

Finally, the COVID‐19 pandemic has resulted in an exponential increase in the use of V‐V ECMO worldwide. As COVID‐19 is a unique syndrome, and many studies and registries are still ongoing, studies reporting on patient treated by V‐V ECMO for COVID‐19‐related ARDS were not included in the current review. The results of the current review can, therefore, not necessarily be extrapolated to patients with COVID‐19, and should be interpreted with caution. Still, the significantly increased use of V‐V ECMO in the past year provides us with the opportunity to improve reporting in ECMO research, enabling researchers to solve the defined ECMO gap, both clinically and academically.

### 4.1 Limitations

The included studies were quite heterogeneous, meaning that not all outcomes were reported in all papers, making it difficult to interpret the results of a true meta‐analysis. Therefore, as illustrated by the levels of heterogeneity, pooled rates should be interpreted with caution. Moreover, a substantial number of initially included studies in the systematic review,^7^, ^46^, ^47^, ^48^, ^49^, ^50^, ^51^, ^52^, ^53^, ^54^ had to be excluded from analysis as they did not report on the most essential outcomes, further defining the ECMO‐gap in reporting on ECMO outcomes. Furthermore, it was challenging to relate mortality to indication as there is no uniformity in reporting of indications and outcomes in ECMO research. Providing specific causes of death is not always possible since autopsies are not routinely performed. In addition, timing of deployment of V‐V ECMO support was not uniformly described in the included studies, as well as duration of time of mortality after weaning, indications, duration of support, weaning strategies and management after weaning. The underreporting of these important features also represents a certain ECMO‐gap in ECMO research, and urges future studies to be more consistent in the reporting of these outcomes. Still, we believe that, despite these potential issues, the main ideas and results of the review do define the patients lost in the ECMO gap, which could be a first step to improved treatment of this specific patient population.

## 5 CONCLUSION

Mortality in V‐V ECMO patients remains high, and the timing of death and its relation to causes of death have been poorly investigated. The current systematic review revealed that a significant number of patients still decease after being successfully weaned from V‐V ECMO support (the *V‐V ECMO gap*), indicating that many patients are still at risk of a dismal prognosis despite recovery of lung function. Underreporting and misreporting of timing and causes of death complicates comprehensive understanding of this phenomenon and represents a second *ECMO research gap*. Future studies should focus on fully, uniformly, and agreed reporting of mortality and outcomes in ECMO research. This could lead to improved understanding of ECMO patients’ courses and outcomes, thereby enhancing their management and decreasing mortality rates on‐ECMO and after weaning.

## CONFLICT OF INTEREST

The authors have no financial disclosure and conflicts of interest to declare.

## AUTHORS’ CONTRIBUTION

SH and MM were responsible for the conception, design, acquisition, analysis, interpretation of the study and its results, and drafting of the manuscript. AM was responsible for acquisition, analysis, and interpretation. FST, AO, MB, LMB, MM, PM, GR, TD, JM, and GB were responsible for the conception, design, and revision of the work. RL was responsible for conception, design, acquisition, analysis, interpretation of the study and its results, and drafting of the manuscript and supervision.

## Supporting information

Table S1
