# Supplementary material for: Defining and understanding the “extra‐corporeal membrane oxygenation gap” in the veno‐venous configuration: Timing and causes of death
Source: Artif Organs. 2021 Sep 7;46(3):349–61. doi: 10.1111/aor.14058 (PMC9293076; doi:10.1111/aor.14058)
Supplement: Supplementary file 1 — Table S1 [file AOR-46-349-s001.docx]

SUPPLEMENTARY DATA

Supplementary Table 1. All studies, including those not reporting on variables warranted for calculation of the V-V ECMO gap.

| **Author** | **Year** | **Patient nr. (ECMO)** | **On ECMO mortality nr.** | **%** | **Weaning rate nr.** | **%** | **In hosp. mort. After weaning nr.** | **%** | **Discharge nr.** | **%** |
| --- | --- | --- | --- | --- | --- | --- | --- | --- | --- | --- |
| Beiderlinden [16] | 2006 | 32 | 5 | 15.6 | 27 | 84.4 | 10 | 31.3 | 17 | 53.1 |
| Benzing* [46] | 1997 | 11 | na | na | na | na | na | na | 7 | 63.6 |
| Bermudez [39] | 2010 | 11 | 3 | 27.3 | 8 | 72.7 | 2 | 18.2 | 6 | 54.5 |
| Bonacchi [17] | 2011 | 30 | 5 | 16.7 | 25 | 83.3 | 3 | 10.0 | 22 | 73.3 |
| Buchner [18] | 2018 | 13 | 1 | 7.7 | 12 | 92.3 | 1 | 7.7 | 11 | 84.6 |
| Cheng [19] | 2016 | 116 | 32 | 27.6 | 84 | 72.4 | 22 | 19.0 | 62 | 53.4 |
| Chimot [20] | 2013 | 52 | 13 | 25.0 | 39 | 75.0 | 12 | 23.1 | 27 | 51.9 |
| Chiu [21] | 2015 | 65 | 28 | 43.1 | 37 | 56.9 | 6 | 9.2 | 31 | 47.7 |
| Combes* [7] | 2018 | 124 | na | na | na | na | na | na | na | na |
| Hong [22] | 2013 | 18 | 1 | 5.6 | 17 | 94.4 | 4 | 22.2 | 13 | 72.2 |
| Kang* [47] | 2017 | 99 | na | na | na | na | na | na | 23 | 23.2 |
| Kon [23] | 2015 | 55 | 13 | 23.6 | 42 | 76.4 | 22 | 40.0 | 20 | 36.4 |
| Kredel* [48] | 2014 | 15 | na | na | na | na | na | na | na | na |
| Kutlesa [24] | 2017 | 40 | 11 | 27.5 | 29 | 72.5 | 4 | 10.0 | 25 | 62.5 |
| Lazzeri* [49] | 2018 | 112 | 50 | 44.6 | 62 | 55.4 | na | na | na | na |
| Lee [25] | 2015 | 45 | 24 | 53.3 | 21 | 46.7 | 13 | 28.9 | 8 | 17.8 |
| Locker* [50] | 2003 | 10 | na | na | 1 | 10.0 | na | na | na | na |
| Luyt* [51] | 2016 | 135 | 53 | 39.3 | 82 | 60.7 | na | na | na | na |
| Messai [26] | 2013 | 17 | 7 | 41.2 | 10 | 58.8 | 1 | 5.9 | 9 | 52.9 |
| Munshi [27] | 2017 | 57 | 18 | 31.6 | 39 | 68.4 | 0 | 0.0 | 39 | 68.4 |
| Nakamura [28] | 2013 | 11 | 3 | 27.3 | 7 | 63.6 | 1 | 9.1 | 6 | 63.6 |
| Ng [29] | 2014 | 31 | 6 | 19.4 | 25 | 80.6 | 1 | 3.2 | 24 | 77.4 |
| Noah [30] | 2011 | 69 | 10 | 14.5 | 59 | 85.5 | 8 | 11.6 | 51 | 73.9 |
| Pappalardo [31] | 2013 | 60 | 19 | 31.7 | 41 | 68.3 | 0 | 0.0 | 41 | 68.3 |
| Peek* [52] | 2010 | 68 | na | na | na | na | na | na | na | na |
| Reeb [32] | 2017 | 8 | 3 | 37.5 | 5 | 62.5 | 1 | 12.5 | 4 | 50.0 |
| Roch [33] | 2014 | 77 | 38 | 49.4 | 39 | 50.6 | 6 | 7.8 | 33 | 42.9 |
| Schmidt* [53] | 2013 | 10 | na | na | na | na | na | na | 6 | 60.0 |
| Song [34] | 2016 | 13 | 4 | 30.8 | 9 | 69.2 | 2 | 15.4 | 7 | 53.8 |
| Staudacher* [54] | 2016 | 175 | na | na | na | na | na | na | na | na |
| Voelker [35] | 2015 | 18 | 7 | 38.9 | 11 | 61.1 | 0 | 0.0 | 11 | 61.1 |
| Wohlfarth [36] | 2014 | 11 | 4 | 36.4 | 7 | 63.6 | 3 | 27.3 | 4 | 36.4 |
| Wu [37] | 2014 | 20 | 4 | 20.0 | 16 | 80.0 | 2 | 10.0 | 14 | 70.0 |
| Wu II [38] | 2017 | 106 | 35 | 33.0 | 71 | 67.0 | 21 | 19.8 | 50 | 47.2 |
| **n=35** | **Total** | **1734** |  | | | | | | | |

* indicates a study which does not report on all outcomes required for definition of the V-V ECMO gap.
